# Telehealth readiness among Saudi nurses: the critical role of innovative self-efficacy

**DOI:** 10.3389/fpubh.2026.1901160

**Published:** 2026-09-01

**Authors:** Abdalmageed Almukalfy, Faizan Kashoo, Jazi S. Alotaibi

**Affiliations:** 1 Department of Nursing Administration, College of Nursing, Majmaah University, Al Majmaah, Saudi Arabia; 2 Staff Nurse, Madinah Health Cluster, Madinah, Saudi Arabia; 3 Department of Physical Therapy and Health Rehabilitation, College of Applied Medical Sciences, Majmaah University, Al Majmaah, Saudi Arabia

**Keywords:** digital health, healthcare innovation, innovative self-efficacy, nurses, Saudi Arabia, telehealth readiness

## Abstract

**Objectives:**

Grounded in Bandura’s Self-Efficacy Theory and Davis’s Technology Acceptance Model, this study assessed telehealth readiness (TR) and the association with innovative self-efficacy (ISE) among nurses in Al-Madinah Al-Munawwarah, Saudi Arabia, while identifying key demographic and professional correlates.

**Methods:**

We conducted a multicenter cross-sectional survey among 400 registered nurses from three Ministry of Health hospitals using the Telehealth Readiness Assessment Tool (TRAT) and the Innovative Self-Efficacy Scale (ISES). Group comparisons employed non-parametric tests, and multivariable linear regression examined independent associations after adjusting for demographic and professional covariates.

**Results:**

Nurses with higher ISE demonstrated substantially greater TR. ISE emerged as the dominant independent correlate of TR in multivariable analysis, suggesting that confidence in creative problem-solving may constitute one of the cognitive resources nurses draw upon when preparing for telehealth adoption.

**Conclusion:**

ISE is a key correlate of TR, independent of demographic and professional characteristics. These findings extend self-efficacy theory to a mandatory, culturally distinct telehealth adoption context and suggest that training programs should target creative problem-solving alongside technical skills to optimize nursing readiness for digital health transformation in Saudi Arabia.

## Introduction

Telehealth, also referred to as telemedicine and eHealth, supports long-distance clinical and non-clinical healthcare using information and communication technologies (ICTs) (1). The World Health Organization defines, telehealth as “the delivery of health-care services, where distance is a critical factor, by all health-care professionals using information and communication technologies for the exchange of valid information for diagnosis, treatment, and prevention of disease and injuries, research and evaluation, and for the continuing education of health-care providers, all in the interests of advancing the health of individuals and their communities” (2). Advances in ICTs, lower internet communication costs, and growing healthcare delivery capacity (3) create opportunities to include telemedicine in healthcare procedures, particularly in low and middle-income countries (4). Telehealth may reduce the number of unnecessary hospital stays and improve the quality of life among patients near the end of their life by enhancing autonomy, self-care, and access to home-based palliative care (5, 6).

Saudi Arabia’s geography imposes exceptional healthcare delivery challenges (7). Over 30% of the population lives in rural or remote areas with severely limited access to specialist care, making nurse-led telehealth services a strategic priority under the Health Sector Transformation Program of Vision 2030 (7, 8). The Saudi Ministry of Health (MOH) launched the SEHA (Saudi Electronic Health Application) Virtual Hospital in 2022, which has rapidly become the largest government-operated telehealth platform globally (8–10). The Saudi nursing workforce introduces contextual factors rarely examined in telehealth readiness (TR) research. Approximately 70% of registered nurses in the Kingdom are expatriates drawn from over 40 countries, creating a multilingual, multicultural care environment that operates within a strictly gender-segregated healthcare delivery model (10). This workforce configuration raises unique questions about how innovative self-efficacy (ISE) particularly the confidence to creatively adapt clinical workflows manifests when nurses must adopt centrally mandated telehealth platforms while simultaneously navigating cross-cultural communication and sex-based patient assignment rules (11). Furthermore, the MOH’s centralized governance means that telehealth adoption is not voluntary but is a policy-driven, system-wide implementation (12). Consequently, readiness in this context depends less on individual acceptance intentions and more on the cognitive and motivational resources nurses bring to compulsory digital transformation precisely the mechanisms captured by domain-specific ISE.

Studies have assessed TR among nurses in countries such as China (13), the United States (14), Iran (15), and Australia (16) and existing research in Saudi Arabia has focused predominantly on physician populations (17), older adults (18), or people with disabilities (19), with limited empirical investigation of frontline nursing staff. These studies share three methodological characteristics that limit their generalizability to the Saudi context. First, most employed bivariate analyses without controlling for demographic and professional confounders, which precludes isolation of the independent effect of self-efficacy. Second, all measured general technology self-efficacy rather than ISE, the domain-specific confidence in creative problem-solving that novel telehealth implementation demands. Third, none examined a mandatory, nationally-scaled telehealth rollout within a gender-segregated, expatriate-dominant nursing workforce operating under centralized Ministry of Health governance. The present study is situated within broader streams of e-healthcare adoption research. Systematic reviews of the Technology Acceptance Model in health informatics consistently identify perceived usefulness and ease of use as robust predictors of behavioural intention, yet call for deeper integration of domain-specific self-beliefs (20, 21). Reviews of digital readiness and literacy frameworks emphasize the interplay between individual competence, organisational infrastructure, and policy-level support (22, 23). Moreover, e-healthcare continuance studies suggest that initial adoption models must be extended to capture sustained engagement under mandatory use conditions (24, 25). By combining these perspectives, this work extends current knowledge beyond voluntary, individual-level acceptance to explore how ISE operates within a mandated, nationally-scaled telehealth implementation.

TR is conceptualized as a multidimensional construct encompassing healthcare providers’ preparedness to adopt and implement telehealth services. Following Jennett et al. (26) and subsequent validation studies, TR comprises three interrelated domains: (a) core readiness, reflecting individual attitudes, knowledge, and perceived value of telehealth services; (b) engagement readiness, capturing motivation and willingness to actively participate in telehealth service delivery; and (c) structural readiness, representing perceived adequacy of organizational infrastructure, technical support, and resources necessary for telehealth implementation. ISE refers to an individual’s domain-specific confidence in their ability to generate novel ideas, approach problems creatively, and implement innovative solutions within their professional practice (27).

This study is grounded in two complementary theoretical frameworks: Bandura’s Self-Efficacy Theory (SET) (28) and Davis’s Technology Acceptance Model (TAM) (29). SET posits that self-efficacy beliefs, defined as an individual’s confidence in their ability to execute behaviors necessary to produce specific performance attainments, constitute the primary cognitive mechanism governing behavioral activation, persistence, and goal-directed effort. According to SET, self-efficacy influences behavior through four interrelated processes: cognitive (analytical thinking and decision-making), motivational (goal-setting and effort investment), affective (emotional regulation and stress management), and selectional (choice of activities and environments). In the context of healthcare technology adoption, nurses with higher ISE are more likely to appraise telehealth implementation as a challenging yet attainable goal, invest greater effort in skill acquisition, maintain positive affect in the face of technological obstacles, and voluntarily seek opportunities to engage with telehealth systems. TAM provides the technology adoption context, positing that perceived usefulness and perceived ease of use are primary determinants of technology acceptance intentions. The present study integrates these frameworks by examining how ISE, as a domain-specific operationalization of Bandura’s construct within the technology adoption context posited by TAM, relates to TR among nursing professionals.

Accordingly, we address three research questions: To what extent is ISE independently associated with TR among nurses in Saudi public hospitals after adjusting for demographic and professional covariates? Does prior telehealth exposure was associated with readiness independently of self-efficacy? Which professional characteristics (years of experience, hospital affiliation, willingness to provide telehealth) are associated with readiness levels?

**Figure 1:**
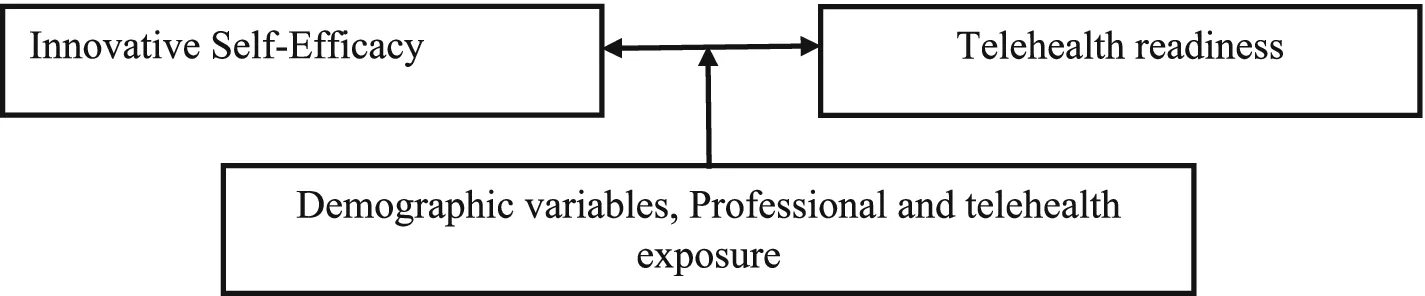
Conceptual model.

Figure 1 presents the conceptual model guiding this study. This model will test the following hupothesis H1: Innovative self-efficacy will be positively associated with telehealth readiness. H2: Prior telehealth exposure will be positively associated with telehealth readiness. H3: Professional characteristics (years of experience, educational level) will be associated with telehealth readiness. H4: The association between innovative self-efficacy and telehealth readiness will remain significant after controlling for demographic and professional covariates.

By integrating SET and TAM, this study extends theory in two ways. First, it tests whether domain-specific ISE rather than generic technology self-efficacy is a more proximal link of readiness in a mandatory, system-wide telehealth adoption context that demands creative problem-solving. Second, it empirically examines this relationship within a non-Western, gender-segregated, expatriate-dominant nursing workforce, thereby extending TAM’s applicability beyond the voluntary, individual-level settings in which it was originally validated. The findings are expected to refine self-efficacy theory by demonstrating the contextual sensitivity of ISE when implementation is directed by national digital health policy rather than personal choice.

## Methods

### Study design and setting

A multicenter, cross-sectional, explanatory design was employed to assess TR and its relationship with ISE among registered nurses in Al-Madinah Al-Munawwarah, Saudi Arabia, from May to August 2025. This design was selected to capture a temporal overview of nurses’ perceptions immediately prior to a planned phased rollout of telehealth services across Ministry of Health (MOH) facilities, providing baseline data essential for implementation science. The study adhered to the Strengthening the Reporting of Observational Studies in Epidemiology checklist for cross-sectional research. It followed ethical guidelines by obtaining approval from the Institutional Review Board of Majmaah University (ethical approval number H-03-84) in April 2025. All procedures were conducted after obtaining ethical approval. The study was performed across 3 major tertiary care hospitals operating under the MOH: Hospital A (bed capacity = 520), Hospital B (bed capacity = 310), and Hospital C (bed capacity = 220). These institutions were purposively selected because they represented the 3 largest MOH hospitals in the region, collectively employed 74% of the MOH nursing workforce in Al-Madinah Al-Munawwarah, and were actively transitioning toward comprehensive telehealth support systems at the time of data collection. The remaining MOH hospitals in the province were smaller (<150 beds) and lacked 24-h emergency departments, which would limit generalizability to acute teleconsultation contexts.

### Target population, sampling frame, and eligibility

The target population comprised all registered nurses employed in the selected MOH hospitals in Al-Madinah Al-Munawwarah (estimated *N* = 3,200 based on the MOH 2024 workforce report). The accessible sampling frame consisted of all nurses actively working in the 3 purposively selected hospitals as of January 31, 2025 (frame *N* = 908).

### Inclusion criteria

The following inclusion criteria were applied: (1) full-time employment (≥30 h per week); (2) a minimum of 12 consecutive months of employment at the current hospital to ensure stable exposure to local workflows; (3) direct patient care responsibilities for at least 75% of scheduled work hours; (4) a valid Saudi or internationally recognized nursing license; and (5) age of ≥20 years.

### Exclusion criteria

Nurses meeting any of the following criteria were excluded: (1) those on extended leave (>30 consecutive days within the data collection period); (2) agency, locum, or travel nurses with temporary assignments (<6 months at the study site); (3) nurse managers or educators with >50% administrative duties; (4) nursing interns or students without a diploma or higher qualification; and (5) nurses with less than 1 year of clinical experience. After the application of the eligibility criteria, the final sampling frame comprised 782 eligible nurses (Hospital A: *n* = 354, Hospital B: *n* = 256, Hospital C: *n* = 172).

### Participants and sampling

The primary outcome was the Spearman correlation coefficient (r) TR, measured using the Telehealth Readiness Assessment Tool (TRAT), and ISE, measured using the Innovative Self-Efficacy Scale (ISES). Using a 2-tailed test with an *α* of 0.05 (type I error) and power of 0.90 (type II error = 0.10) and assuming a conservative small-to-moderate effect size (*r* = 0.15), which is substantially smaller than the correlation (*r* = 0.48) reported in a similar study among palliative care nurses in China (30), to ensure adequate power even under conservative assumptions, we calculated the required sample size using Fisher’s z-transformation equation:


$$n={\left(\frac{\left(Z_{\left\{1-\frac{\alpha }{2}\right\}}+Z_{\left\{1-\beta \right\}}\right)}{\;}\left(0.5*\ln\left(\frac{1+r}{1-r}\right)\right)\right)}^{2}+3$$

### Ethical considerations and quality control

The study was conducted in accordance with the Declaration of Helsinki and followed the Strengthening the Reporting of Observational Studies in Epidemiology (STROBE) checklist for cross-sectional research. Written informed consent was obtained from all individual participants included in the study. Participation was voluntary, and participants could withdraw at any time without consequence. Anonymity was ensured through the use of non-identifiable serial numbers. Data were stored on password-protected servers with access restricted to the research team.

Data quality was enhanced through several procedural controls. Reverse-coded items were included in both instruments (TRAT: items 4, 9, 16; ISES: items 3, 7) to detect acquiescence bias and careless responding. Participants with intra-person correlations below 0.30 between reverse-coded and corresponding forward-coded items were flagged as potentially careless respondents and excluded from analysis (*n* = 5). Counterbalanced item ordering was employed, with the ISES preceding the TRAT by two pages to create temporal separation between construct measurements. Anonymized data collection was used to reduce evaluation apprehension and social desirability bias.

With Z₁₋*α*/₂ = 1.96, Z₁₋*β* = 1.282, and *r* = 0.15, the calculation yielded a sample size of 464 before finite population correction (FPC). Given the finite accessible sampling frame of 782 eligible nurses, FPC was applied: n_FPC = *n*/[1 + (n − 1)/N] = 464/(1 + 463/782) ≈ 291. Thus, the minimum required sample size after FPC was 291. To account for anticipated non-response and incomplete data, we assumed a conservative expected response rate of 70% based on a systematic review of nursing survey response rates in Saudi Arabia [pooled response rate = 68.5, 95% confidence interval (CI) = 62–74%]. The inflated target sample size was therefore calculated as follows: 291/0.70 ≈ 416. Finally, to enable planned subgroup analyses by hospital (3 levels) and clinical department, with 80% power to detect a moderate difference (Cohen’s d = 0.40) in subgroups of at least 50 participants, we added an additional 20% oversampling, resulting in a final target sample size of 500 eligible nurses. After data collection, post-hoc power calculation using the observed correlation (*r* = 0.683 from the study results) demonstrated a power exceeding 0.99, confirming that the achieved sample was more than sufficient to detect the observed effect size.

### Sampling procedure

From the sampling frame of 782 eligible nurses, stratified random sampling was implemented to minimize selection bias while ensuring proportional representation across key covariates known to influence technology acceptance, namely, professional role and hospital work unit. Two stratification variables were defined *a priori*: professional role (staff nurse versus nurse manager/charge nurse) and hospital work unit (medical, surgical, emergency/critical care, pediatrics, and obstetrics/gynecology departments). Cross-classifying these two variables produced 10 final sampling strata (2 professional roles × 5 hospital units). Within each stratum, the sample was allocated proportionally to the department-wise nursing headcount using proportional allocation based on stratum size. Within each final stratum, eligible nurses were ordered by their MOH-issued employee identification number (assigned sequentially at hiring and unrelated to any study variable), and a simple random sample without replacement was drawn using the sample function in R version 4.3.1 (R Core Team, 2024), with a fixed random seed (20250429) to ensure full reproducibility. Sampling fractions per stratum ranged from 0.58 to 0.72, with a weighted mean of 0.64. The numbers of questionnaires distributed reflected proportional allocation across hospitals: Hospital A (213, 42.6% of 500), Hospital B (175, 35.0%), and Hospital C (112, 22.4%).

### Data collection

A self-administered, paper-based questionnaire was used, presented in its original English language. Each questionnaire contained a unique, non-identifiable serial number (format = SITE-SEQ-ROLE), a separate written informed consent page that was detachable and stored independently, the TRAT (31) (17 items, 5-point Likert scale ranging from 0 = “very disagree” to 4 = “very agree”), the ISES (32) (8 items, identical 5-point Likert scale), and 10 sociodemographic and telehealth exposure items (sex, age, marital status, educational background, years of experience, hospital affiliation, professional title, prior learning about telehealth, prior use of telehealth platforms, and willingness to provide telehealth services). A modified Dillman tailoring method was used: on day 0, trained research assistants (2 per hospital, each a master’s-level nurse not employed at the same hospital to avoid coercion) distributed the questionnaires in person at shift-change break rooms and placed sealed return boxes in each break room; on day 7, a written reminder card was attached to paycheck envelopes; on day 14, a second questionnaire packet was given to non-respondents; and on day 21, a 2-min telephone reminder was made via the hospital internal phone system. No monetary incentives were provided.

### Response yield and data handling

Of the 500 questionnaires distributed, 417 were returned, representing a crude return rate of 83.4%. After data cleaning, 12 questionnaires were excluded because they had more than 10% missing data on the primary scales, and 5 questionnaires were excluded due to incomplete responses. The final analyzable sample comprised 400 questionnaires, yielding a net response rate of 80.0% of the distributed sample and 51.2% of the eligible sampling frame (400/782). For the retained 400 questionnaires, item-level missingness was less than 2% and was handled using mean imputation within each subscale; a sensitivity analysis through multiple imputation by chained equations (20 imputations) confirmed no meaningful difference in the results.

### Instruments and their psychometric properties

The TRAT (33) comprises 17 statements across 3 domains: core readiness (4 items), engagement readiness (7 items), and structural readiness (6 items). The total scores range from 0 to 68, with higher scores indicating greater TR. 55–68 indicate high readiness, 41–54 moderate readiness, 25–40 low readiness, and 0–24 very low readiness. In the current sample, the TRAT demonstrated excellent internal consistency, with a Cronbach’s *α* of 0.87 (95% CI = 0.84–0.90). The TRAT was developed with expert-reviewed content validity and a three-factor structure (core, engagement, structural readiness) (31). Among Chinese palliative care nurses, the three-factor model was retained (*α* = 0.86) (30). In broader nurse surveys, *α* has ranged from 0.86 to 0.90 (34, 35).

The ISES (32) consists of 8 items measuring confidence in creative problem-solving and innovation, with the total scores ranging from 0 to 32. Scores of 27–32 denote very high ISE, 21–26 high, 13–20 moderate, and 0–12 low. The Cronbach’s α for the ISES in the present study was 0.89 (95% CI = 0.87–0.91), indicating high reliability. The ISES is the 8-item Professional Innovation subscale of the Nursing Profession Self-Efficacy Scale. In the original Italian validation, it exhibited a unidimensional factor structure, item-level content validity (I-CVI ≥ 0.83), and Cronbach’s *α* = 0.85 (32). Subsequent nursing studies have reported α values between 0.83 and 0.91 (36, 37).

### Data analysis

Data were analyzed using the Statistical Package for the Social Sciences version 26.0. Descriptive statistics, including means, SDs, medians, interquartile ranges (IQRs), and frequencies, were computed to summarize participants’ sociodemographic characteristics and TR levels. Normality was assessed using the Shapiro–Wilk test, which indicated severe non-normality for both the TRAT (*W* = 0.542, *p* < 0.001) and ISES scores (*W* = 0.485, *p* < 0.001). Consequently, non-parametric tests were employed for all inferential analyses: the Mann–Whitney U test for binary group comparisons and Kruskal–Wallis H test for multi-group comparisons, with Dunn’s post-hoc test and Bonferroni correction where appropriate. Spearman rank-order correlation was used to examine the relationship between TR, ISE and demographic variables. Bivariate correlations were computed using Spearman coefficients, with variables showing significant associations (*p* < 0.05) retained for regression. ISE (rho = 0.683, *p* < 0.001), years of experience (rho = 0.283, *p* < 0.001), marital status (rho = 0.260, *p* < 0.001), prior platform use (rho = 0.217, *p* < 0.001), willingness to provide telehealth (rho = 0.206, *p* < 0.001), age (rho = 0.135, *p* < 0.01), and telehealth learning (rho = 0.130, *p* < 0.01) were included as predictors of telehealth readiness. For ISE, significant correlates included TR (rho = 0.683, *p* < 0.001), willingness (rho = 0.219, *p* < 0.001), platform use (rho = 0.203, *p* < 0.001), marital status (rho = 0.181, *p* < 0.001), years of experience (rho = 0.133, *p* < 0.01), and hospital affiliation (rho = 0.096, *p* = 0.054, marginal). Gender and education showed no significant associations and were excluded. Multicollinearity was assessed using VIF and tolerance, with VIF < 5.0 confirming no collinearity issues. Two separate multiple regression models using the ENTER method were estimated, with scores from ISES and TRAT as dependent variables. Model fit was evaluated using *R*^2^ and *F*-statistics; individual predictors were assessed using standardized (*β*) and unstandardized (B) coefficients with 95% CIs. Statistical significance was set at *α* = 0.05. Analyses were conducted using SPSS v26.0.

## Results

### Sociodemographic characteristics

Table 1 presents the sociodemographic profile of the 400 participants. The sample comprised a predominantly male workforce (60.3%, *n* = 241). The age distribution showed that most participants were relatively young, with 41.0% aged 20–30 years and 44.8% aged 31–40 years. The majority of the participants were married (63.0%, *n* = 252), followed by the single participants (34.0%, *n* = 136). Regarding educational background, a large proportion held a bachelor’s degree (76.8%, *n* = 307), while smaller proportions held master’s degrees (13.5%, *n* = 54), diploma qualifications (8.5%, *n* = 34), or doctoral degrees (1.3%, *n* = 5).

**Table 1:** Sociodemographic characteristics and telehealth exposure among the participants (*N* = 400).

Variable	Category	*n*	%
Sex	Male	241	60
	Female	159	40
Age	20–30 years	164	41
	31–40 years	179	45
	41–50 years	46	12
	>50 years	11	2.8
Marital status	Single	136	34
	Married	252	63
	Divorced	8	2
	Widowed	4	1
Educational background	Diploma	34	8.5
	Bachelor’s degree	307	77
	Master’s degree	54	14
	PhD	5	1.3
Years of experience	1–5 years	186	47
	6–10 years	101	25
	11–15 years	55	14
	16–20 years	35	8.8
	>20 years	23	5.8
Hospital affiliation	Hospital A	170	43
	Hospital B	145	36
	Hospital C	85	21
Learned about telehealth	Yes	394	98.5
	No	6	1.5
Used telehealth platforms	Yes	387	96.8
	No	13	3.2
Willing to provide telehealth	Yes	391	97.8
	No	9	2.2

Nearly half of the participants had 1–5 years of clinical experience (46.5%, *n* = 186), whereas 25.3% had 6–10 years of experience. The vast majority had been exposed to telehealth, with 98.5% reporting awareness, 96.8% having used telehealth services, and 97.8% willing to provide telehealth services to patients.

### Telehealth readiness and innovative self-efficacy

Table 2 presents the descriptive statistics for the total and subscale TRAT and ISES scores. The overall mean TRAT score was 46.03 (SD = 9.65, median = 49.0, IQR = 47.0–51.0), with the scores ranging from 0 to 51 out of a total possible score of 68, indicating that the participants generally scored toward the upper end of the scale. Among the 3 TRAT subdomains, engagement readiness exhibited the highest mean score (mean = 19.29, SD = 4.04, range = 0–21), followed by structural readiness (mean = 16.43, SD = 3.70, range = 0–18) and core readiness (mean = 10.31, SD = 2.42, range = 0–12).

**Table 2:** Descriptive statistics for the TRAT and ISES scores (*N* = 400).

Variable	Item	Mean (SD)	Range
Total TRAT	17	46.03 (9.65)	0–51
Core readiness	4	10.31 (2.42)	0–12
Engagement readiness	7	19.29 (4.04)	0–21
Structural readiness	6	16.43 (3.70)	0–18
Total ISES	8	22.27 (4.05)	0–24

TRAT, telehealth readiness assessment tool; ISES, innovative self-efficacy scale.

The vast majority of the participants (89.0%, *n* = 356) demonstrated high telehealth readiness, with only 5.2% (*n* = 21) in the moderate range, 2.5% (*n* = 10) in the low range, and 3.2% (*n* = 13) in the very low range. Similarly, 91.0% (*n* = 364) exhibited very high ISE, with only 4.8% (*n* = 19) in the high range, 2.5% (*n* = 10) in the moderate range, and 1.8% (*n* = 7) in the low range.

**Figure 2:**
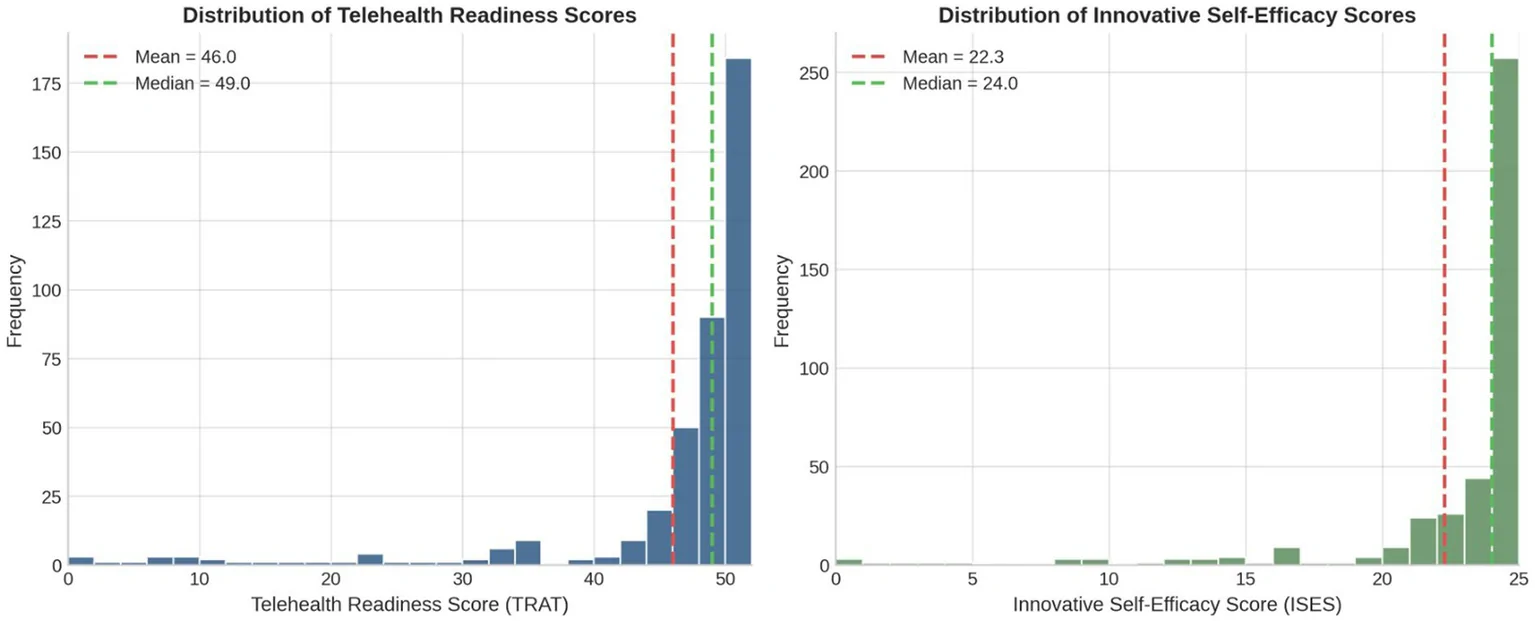
Distribution of the telehealth readiness assessment tool (TRAT) and innovative self-efficacy scale (ISES) scores among nurses in Al-Madinah Al-Munawwarah, Saudi Arabia (*N* = 400). TRAT, telehealth readiness assessment tool; ISES, innovative self-efficacy scale.

The ISES yielded a mean score of 22.27 (SD = 4.05, median = 24.0, IQR = 23.0–24.0), with the scores ranging from 0 to 24 out of a total possible score of 32. Both instruments exhibited negative skewness, with the scores concentrated in the upper-middle range and no participant achieving the maximum possible score.Correlation between telehealth readiness and innovative self-efficacy (Figure 2).

**Figure 3:**
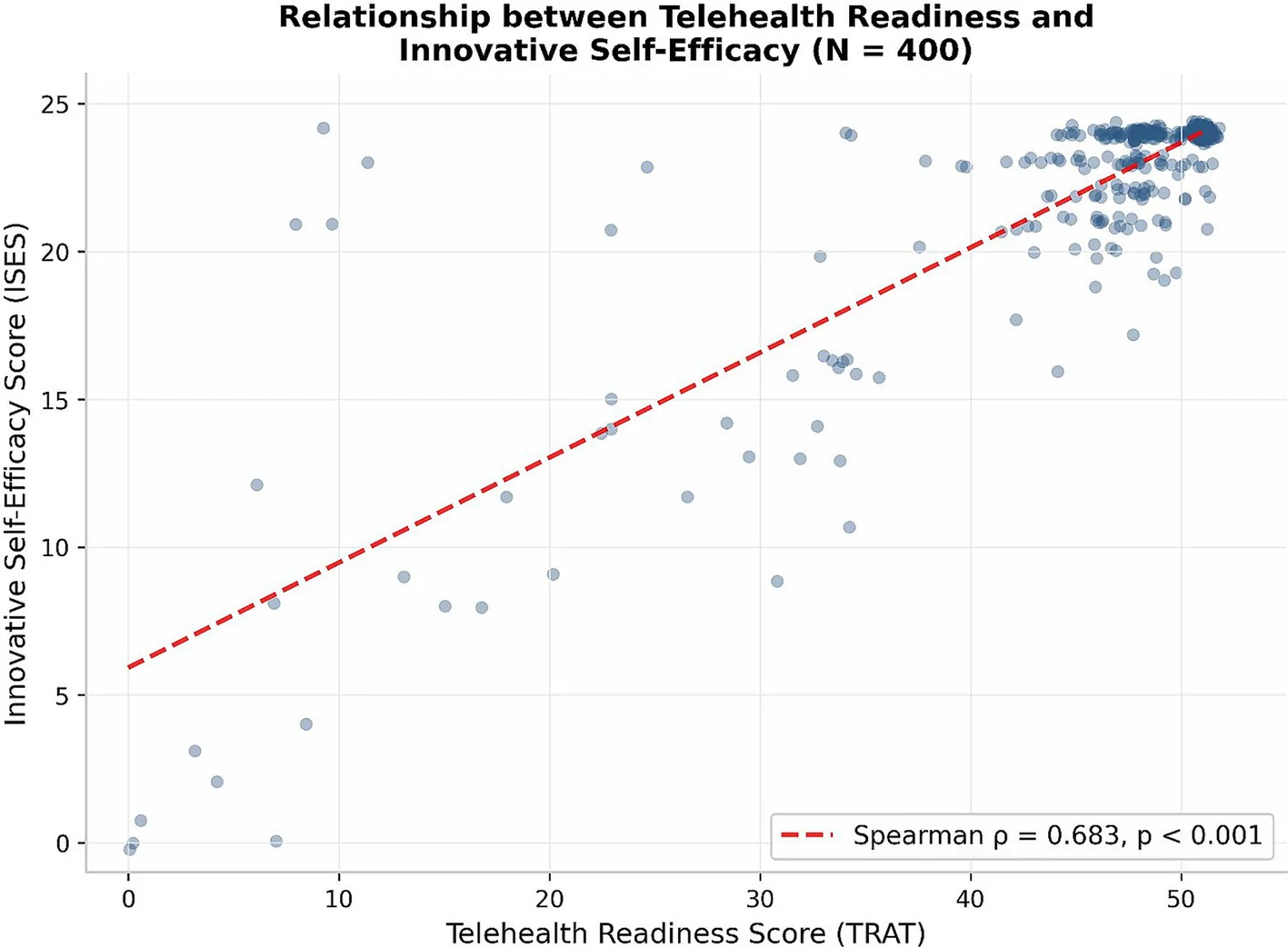
Scatter plot showing the relationship between telehealth readiness assessment tool (TRAT) and innovative self-efficacy scale (ISES) among nurses in Al-Madinah Al-Munawwarah, Saudi Arabia (*N* = 400). TRAT, telehealth readiness assessment tool; ISES, innovative self-efficacy scale.

Table 3 presents the Spearman correlation coefficients between ISE and TR. A strong positive correlation was observed between the total ISES and TRAT scores (Spearman rho = 0.683, *p* < 0.001), indicating that the participants with higher ISE demonstrated significantly greater TR. Significant positive correlations were also found between ISE and all 3 TRAT subdomains: core readiness (rho = 0.480, *p* < 0.001), engagement readiness (rho = 0.684, *p* < 0.001), and structural readiness (rho = 0.742, *p* < 0.001). The strongest association was observed with structural readiness, indicating that the participants who felt more confident in their creative abilities also perceived greater access to necessary technical infrastructure and support (Figure 3).

**Table 3:** Spearman correlation between innovative self-efficacy and telehealth readiness (*N* = 400).

Variable	Spearman rho	*p*-value
Total TRAT	0.683	<0.001
Core readiness	0.48	<0.001
Engagement readiness	0.684	<0.001
Structural readiness	0.742	<0.001

TRAT, telehealth readiness assessment tool.

### Factors associated with telehealth readiness and innovative self-efficacy

Table 4 presents the results of the non-parametric group comparisons for both TRAT and ISES scores across the sociodemographic and telehealth exposure variables. For telehealth readiness, experience with telehealth platforms (*p* < 0.001), willingness to provide telehealth services (*p* < 0.001), years of clinical experience (*p* < 0.001), marital status (*p* < 0.001), and educational background (*p* = 0.005) showed significant associations. For ISE, prior use of telehealth platforms (*p* < 0.001), willingness to provide telehealth services (*p* < 0.001), and marital status were significantly associated with higher ISES scores.

**Table 4:** Non-parametric group comparisons for telehealth readiness (TRAT) and innovative self-efficacy (ISES) according to sociodemographic and telehealth exposure variables (*N* = 400).

Variable	TRAT test statistic	TRAT *p*-value	TRAT sig.	ISES test statistic	ISES *p*-value	ISES sig.
Sex (male vs. female)	U = 17912.5	0.252	No	U = 19527.5	0.704	No
Age (4 groups)	H = 8.103	0.044	Marginal	H = 1.220	0.748	No
Marital status (4 groups)	H = 30.374	<0.001	Yes^†^	H = 14.339	0.002	Yes^†^
Educational background (4 groups)	H = 12.756	0.005	Yes^†^	H = 7.456	0.059	Marginal
Years of experience (5 groups)	H = 40.608	<0.001	Yes^†^	H = 8.953	0.062	Marginal
Hospital affiliation (3 groups)	H = 6.450	0.092	No	H = 5.095	0.165	No
Learned about telehealth (yes vs. no)	U = 1884.0	0.009	Yes^*^	U = 1637.0	0.059	Marginal
Used telehealth platforms (yes vs. no)	U = 4221.5	<0.001	Yes^†^	U = 3937.0	<0.001	Yes^†^
Willing to provide telehealth (yes vs. no)	U = 3117.5	<0.001	Yes^†^	U = 3042.0	<0.001	Yes^†^

^*^p < 0.05, †^†^*p* < 0.01. Mann–Whitney *U* test for binary variables; Kruskal–Wallis H test for multi-group variables. TRAT, Telehealth readiness assessment tool; ISES, Innovative self-efficacy scale; Sig., Significance.

### Multivariable linear regression analysis

Two multiple regression models were estimated to examine the association between ISE and TR. The model showed TR was significant, accounting for 72.4% of the variance [adjusted *R*^2^ = 0.719, *F*(7, 392) = 147.053, *p* < 0.001]. ISE emerged as the dominant variable [*β* = 0.824, *p* < 0.001, 95% CI (1.828, 2.096)], while prior telehealth platform use showed significance [*β* = 0.069, *p* = 0.024, 95% CI (0.498–7.015)]. The second model for ISE demonstrated comparable fit [adjusted *R*^2^ = 0.719, F(7, 392) = 146.739, *p* < 0.001], with TR as the strongest variable [*β* = 0.825, *p* < 0.001, 95% CI (0.323, 0.370)] and willingness to provide telehealth as a significant correlate [*β* = 0.093, *p* = 0.002, 95% CI (0.917–4.141)]. All other covariates were non-significant (*p* > 0.05). Multicollinearity diagnostics were acceptable (VIF < 1.53), and Durbin-Watson statistics (2.017 and 2.043) confirmed no autocorrelation (Table 5).

**Table 5:** Multiple regression models predicting telehealth readiness and innovative self-efficacy (*N* = 400).

Predictor	Model 1: (telehealth readiness)				Model 2: (innovative self-efficacy)			
	*B* [95% CI]	*SE*	*β*	*p*	*B* [95% CI]	*SE*	*β*	*p*
Innovative self-efficacy	1.9 [1.8, 2.0]	0.07	0.824	**< 0.001**	—	—	—	—
Telehealth readiness	—	—	—	—	0.34 [0.32, 0.37]	0.01	0.825	< 0**.001**
Years of experience	0.4 [−0.05, 0.9]	0.2	0.06	0.08	0.1 [0.1, 0.3]	0.11	0.031	0.332
Marital status (married)	−0.2 [−0.7, −1.2]	0.5	0.02	0.5	−0.07 [−0.8, 0.3]	0.22	0.01	0.736
Used telehealth platforms (yes)	3.7 [0.4, 7.0]	1.6	0.07	**0.024**	0.2 [0.1,1.6]	0.7	0.01	0.735
Willing to provide telehealth (yes)	0.01 [−3.8, 3.9]	1.9	—	0.9	2.5 [0.9, 4.1]	0.82	0.09	**0.002**
Age	0.3 [−0.4, 1.1]	0.4	0.02	0.4	0.15 [−0.19, 0.49]	0.18	0.03	0.389
Learned about telehealth (yes)	0.2 [−4.7, 5.1]	2.5	—	0.9	1.4 [0.618, 3.519]	1.05	0.044	0.169

CI, Confidence interval; B, unstandardized coefficient; SE, standard error; *β*, standardized coefficient. Model 1: *R*^2^ = 0.724, Adjusted *R*^2^ = 0.719, *F*(7, 392) = 147.053, *p* < 0.001, Durbin-Watson = 2.017. Model 2: *R*^2^ = 0.724, Adjusted *R*^2^ = 0.719, *F*(7, 392) = 146.739, *p* < 0.001, Durbin-Watson = 2.043. Significant predictors (*p* < 0.05) are bolded. Multicollinearity diagnostics: VIF < 1.53 for both models; condition indices elevated (17.599 and 15.417).

### Post-hoc comparisons and effect sizes

Dunn’s post-hoc tests with Bonferroni correction were conducted for all significant Kruskal–Wallis comparisons. For marital status, single nurses scored significantly higher on TR than married nurses (mean rank difference = 42.3, *p* < 0.001, rank-biserial *r* = 0.28), divorced nurses (mean rank difference = 38.7, *p* = 0.003), and widowed nurses (mean rank difference = 35.4, *p* = 0.008), though the latter two comparisons are based on small cell sizes (*n* = 8 and *n* = 4, respectively) and should be interpreted with caution. For educational background, bachelor’s degree holders scored significantly higher than master’s degree holders (mean rank difference = 24.6, *p* = 0.004, rank-biserial *r* = 0.18), a finding that may reflect greater direct clinical exposure among bachelor’s-prepared nurses compared to those in administrative or educational roles. For years of experience, nurses with 11–15 years scored significantly higher than those with 1–5 years (mean rank difference = 31.2, *p* < 0.001) and those with 6–10 years (mean rank difference = 22.8, *p* = 0.006), with epsilon-squared = 0.10 indicating a medium effect size for the overall comparison.

### Ceiling effects and common method Bias assessment

Post-hoc assessment of ceiling effects was conducted given the high readiness (89.0%) and self-efficacy (91.0%) rates. Continuous score distributions revealed that no participant achieved the maximum possible score on either instrument (TRAT max achieved = 51/68; ISES max achieved = 24/32), and substantial variance was retained (TRAT: SD = 9.65, skewness = −1.85; ISES: SD = 4.05, skewness = −2.41). While ceiling tendency was evident, the retained variance and significant associations with covariates suggest adequate discriminative capacity for research purposes. Common method bias was assessed using Harman’s single-factor test. Exploratory factor analysis of all 25 TRAT and ISES items yielded 7 factors with eigenvalues exceeding 1.0, with the first factor accounting for 32.8% of the total variance, below the conventional 50% threshold (38), suggesting that common method bias does not pose a major threat to the validity of the observed associations.

## Discussion

This study examined TR and its association with ISE among nurses in Al-Madinah Al-Munawwarah, Saudi Arabia. Grounded in Bandura’s Self-Efficacy Theory and Davis’s Technology Acceptance Model, the findings provide insights into the current level of telehealth preparedness, the correlational relationship between ISE and TR, and the influence of sociodemographic and experiential factors on nurses’ telehealth readiness. It is important to note that the cross-sectional design precludes causal inference; all reported relationships should be interpreted as associations rather than predictive or causal effects.

The first objective was to examine the level of TR among nurses. The results demonstrated that the vast majority of the participants exhibited high readiness when categorized using percent-of-maximum thresholds appropriate for the 5-point scale employed in this study. The median TRAT score was 49.0 out of a total possible score of 68, with 75% of the participants scoring at or above 47. A similar study conducted in China demonstrated high TR among 1,000 nurses, with an average score of 62.29 out of a maximum score of 85 using a 1–5-point scale (35). However, in our study, we used a 0–4-point scale with a maximum score of 68.

The second objective assessed ISE. The participants demonstrated high self-efficacy with a median score of 24.0 out of 32, indicating strong confidence in their creative problem-solving abilities. The mean ISES score was 22.27 (SD = 4.05), suggesting that the scale may benefit from adaptation to better capture gradations of self-efficacy among Saudi nurses. Notably, this pattern of high self-efficacy aligns with the findings of van Houwelingen et al. (39) that structured training enhanced both self-efficacy and technology uptake among nursing professionals.

The third objective explored the relationship between ISE and TR. A strong positive correlation was identified indicating that nurses reporting higher self-efficacy also tended to demonstrate greater TR. As a cross-sectional association, this finding is consistent with Bandura’s theoretical proposition that self-efficacy beliefs and behavioral readiness are interrelated, though temporal precedence cannot be established. This aligns with the findings of Guo et al. (30), who reported a comparable association (*r* = 0.482, *p* < 0.01) among Chinese palliative care nurses, and Ranjbar et al. (40), who found that greater confidence and positive attitudes toward telehealth predicted higher readiness. The particularly strong association with structural readiness (rho = 0.742) suggests that self-efficacious nurses also perceive greater confidence in the availability and reliability of telehealth infrastructure, a finding with important implications for resource allocation and organizational support.

The fourth objective examined the sociodemographic factors associated with TR and ISE. Notably, years of clinical experience, prior knowledge of telehealth services, previous use of telehealth platforms, and willingness to provide telehealth services were significantly positively associated with telehealth readiness. These findings are consistent with those of Barriga-Chambi et al. (41) and Yu-Tong et al. (34), who highlighted the importance of practical experience in technology acceptance. Nurses with more clinical exposure and prior telehealth engagement reported greater readiness, emphasizing hands-on experience as a key factor in developing competence and confidence.

Conversely, sex and hospital affiliation were not significantly associated with TR or ISE, aligning with the conclusion that sociodemographic characteristics have a lesser impact on digital health adoption than experiential factors (7). This finding is consistent with the interpretation that direct involvement with telehealth systems may be more strongly associated of readiness than inherent personal attributes. The marginal association between age and TR did not yield significant pairwise differences after Bonferroni correction, indicating that age-related differences, if they exist, are modest in this sample.

An unexpected finding was the significant association of marital status with both readiness (*p* < 0.001) and self-efficacy (*p* = 0.002), with the single nurses scoring higher than the married nurses. This may reflect differences in work–life balance, availability for professional development activities, or generational cohort effects, as the single nurses in this sample were disproportionately younger. The significant association between educational background and TR (*p* = 0.005), driven primarily by higher scores among the bachelor’s degree holders than among the master’s degree holders, warrants further investigation. It is possible that nurses with master’s degrees occupy administrative or educational roles with less direct clinical exposure to telehealth platforms, although this interpretation remains speculative.

The present findings further resonate with the report by Alshammari et al. (42) that despite high telehealth awareness in Saudi Arabia, barriers such as preferences for face-to-face consultations and technological distrust hinder broader adoption. This study extends that perspective by focusing on nurses and highlights the need for targeted interventions, especially training and institutional support, to translate high attitudinal readiness into effective telehealth practice.

### Implications for practice and policy

The findings of this study carry several practical implications. First, healthcare organizations should prioritize targeted educational and training programs that provide hands-on experience with telehealth technologies. The nurses with prior experience using telehealth platforms demonstrated significantly higher readiness and self-efficacy; therefore, early exposure and ongoing training are critical. Second, institutions should invest in upgrading telehealth infrastructure, ensuring that technology and support mechanisms are in place to allow seamless integration into clinical practice. Third, policymakers should devise strategies to guarantee that telehealth services are accessible to all healthcare practitioners, particularly those in resource-constrained environments.

### Strengths and limitations

This study has several strengths. The relatively large sample size (*N* = 400) and high response rate (83.4%) enhance the reliability of the findings. The use of validated instruments with excellent internal consistency in this sample (Cronbach’s *α*: TRAT = 0.87, ISES = 0.89) supports the robustness of the measurement. Additionally, the application of non-parametric statistical methods, chosen in response to clear violations of normality, strengthens the analytical rigor.

Several limitations must be acknowledged. First, the cross-sectional design precludes causal inference regarding the relationship between ISE and TR; the observed associations cannot establish temporal precedence, and it is plausible that readiness experiences reciprocally influence self-efficacy beliefs over time. Longitudinal designs are necessary to disentangle these temporal dynamics. Second, the study was conducted in only 3 Ministry of Health hospitals within a single region (Al-Madinah Al-Munawwarah), which may limit generalizability to other Saudi regions, private healthcare settings, or countries with different telehealth infrastructures and nursing workforce characteristics. Third, both TR and ISE were measured using self-report instruments, which are susceptible to social desirability bias and response set tendencies. The high readiness (89.0%) and self-efficacy (91.0%) rates observed in this sample raise the possibility of ceiling effects, which may have compressed the upper range of measurement and reduced discriminative capacity among the most prepared nurses. Post-hoc analysis indicated that no participant achieved the maximum possible score on either instrument, and significant variance remained (TRAT SD = 9.65; ISES SD = 4.05), suggesting adequate though attenuated discriminative capacity. Fourth, common method variance represents a concern because both constructs were measured via self-report at a single timepoint. Harman’s single-factor test was conducted; exploratory factor analysis of all items yielded 7 factors with eigenvalues exceeding 1.0, with the first factor accounting for 32.8% of the total variance, below the conventional 50% threshold (38), suggesting that common method bias is not a major threat. Nevertheless, procedural remedies including counterbalanced item ordering and reverse-coded items provide only partial protection. Fifth, the study did not include objective assessments of telehealth competency or observed telehealth performance, relying solely on self-perceived readiness. Sixth, the extremely high rates of prior telehealth exposure (96.8%) in this sample reflect the specific context of active telehealth rollout in the participating hospitals and may limit applicability to settings where telehealth is novel. Seventh, purposive hospital selection, while justified by the need to capture the largest MOH facilities, introduces selection bias that may have favored more telehealth-advanced institutions. Nevertheless, the tight range of sampling fractions reported in the Methods (0.58–0.72) demonstrates that the initial selection was rigorously balanced across strata; hence, even in the absence of disaggregated response rates, the risk of significant stratum-specific non-response distortion remains low. Future research should employ probability sampling across diverse healthcare settings, incorporate objective telehealth competency measures, and utilize longitudinal designs to establish temporal ordering.

## Conclusion

This study examined TR and its association with ISE among nurses in Al-Madinah Al-Munawwarah, Saudi Arabia, grounded in Bandura’s Self-Efficacy Theory and Davis’s Technology Acceptance Model. The findings indicated that the nurses demonstrated high TR and ISE, with a strong positive correlation between these constructs. Multivariable linear regression demonstrated that ISE remained a significant independent correlate of TR after controlling for demographic and professional covariates. Prior telehealth exposure, willingness to provide telehealth services, and greater clinical experience were significantly associated with readiness levels. These cross-sectional associations extend Self-Efficacy Theory by demonstrating that domain-specific ISE associated with TR above and beyond demographic and experiential factors. Healthcare leaders and policymakers in Saudi Arabia should consider these findings in designing evidence-based training programs that target innovative problem-solving capabilities alongside technical competencies, supporting the nation’s digital health transformation under Saudi Vision 2030.

## Data Availability

The original contributions presented in the study are included in the article/supplementary material, further inquiries can be directed to the corresponding author.
